# Bacterial Community Composition and Functional Potential of the Kleptoplastic Sea Slug *Elysia papillosa*

**DOI:** 10.3390/biom16060918

**Published:** 2026-06-20

**Authors:** Jada L. Brown, Padmanabhan Mahadevan, Michael Middlebrooks

**Affiliations:** 1 Department of Biology, The University of Tampa, Tampa, FL 33606, USA; 2 Department of Biological Sciences, Florida Atlantic University, Boca Raton, FL 33431, USA

**Keywords:** sea slug, sacoglossan, shotgun, metagenomics, bacterial diversity, kleptoplasty, *Elysia papillosa*

## Abstract

Certain sacoglossan sea slugs, often known as “solar-powered sea slugs”, are a group of marine gastropods that have the unique ability to photosynthesize by stealing functional chloroplasts from algae. The sacoglossan *Elysia papillosa* can maintain functional chloroplasts for up to two weeks after feeding. The microbiome of these slugs may play a crucial role in their metabolism, immunity, development, but more importantly their photosynthesis. Shotgun metagenomic sequencing was conducted on four samples of *E. papillosa* in order to characterize their microbiome. Sequences were classified and relative abundance was quantified with Centrifuger and functional data was examined using SqueezeMeta. Bacteria were analyzed by taxonomic groups and hypothesized function to the sea slug was determined with literature analysis. All samples were dominated by phyla Actinomycetota, Bacillota, Patescibacteriota, and Pseudomonadota. The presence of the phyla Bacteroidota and Bacillota was notable in all samples, which contain species known to produce enzymes that break down polysaccharides. It is possible that these bacteria could assist in degradation of the polysaccharide xylan found in the cell walls of *Penicillus*, the algal food source of *E. papillosa*. One species that was found in all samples was *Cutibacterium acnes* which has been shown to be an important component of the gut microbiota in other marine invertebrates and may provide the host with vitamin B12 and other beneficial nutrients. Many of these bacteria may be opportunistic rather than commensal. As a result, more research is required to describe the interactions between the slug and its microbiome, but this preliminary report provides a valuable starting point for identifying the microbiome make-up to further understanding of these relationships.

## 1. Introduction

Heterobranch sea slugs are several diverse groups of marine gastropods which have convergently evolved the loss of their shell [1]. These organisms that have evolutionarily lost a protective shell have implemented other adaptations for survival [2,3,4,5], some with the assistance of symbionts. One type of adaptation allowed for by the absence of a shell is kleptoplastic herbivory, which involves the sea slug taking functional chloroplasts from an algal food source and using them to photosynthesize. Kleptoplasty is utilized by some, but not all, sea slugs in the Sacoglossa [6,7,8]. For example, some sacoglossans will store the chloroplasts in their tissues and, in species such as *Caliphylla mediterranea* and *Placida dendritica*, the chloroplasts are non-functional for photosynthesis but alter the body pigmentation to greener tones allowing for camouflage on their algal food source [9,10]. In other species, the stored chloroplasts are functional providing energy to the sea slug through nutrient byproducts of photosynthesis, allowing the organism to survive many months without any additional food source with the duration depending on the species of sacoglossan and their diet [11,12,13]. The ability of these organisms to gather and sustain functional chloroplasts in their tissues raises intriguing possibilities of important interactions with symbiotic microorganisms to assist in these complex processes.

**Figure 1:**
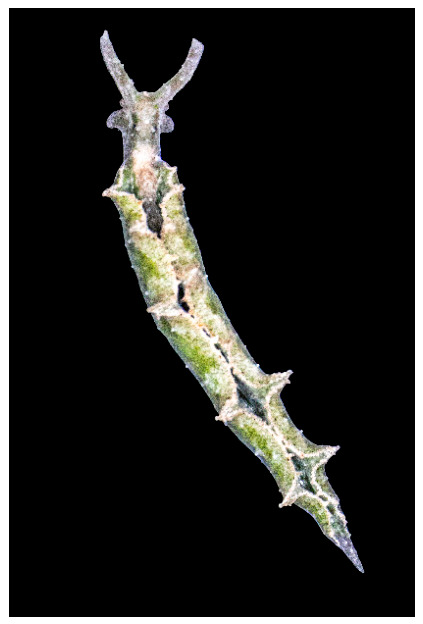
Photograph of *Elysia papillosa* collected from the algae *Penicillus capitatus* from Tarpon Springs, FL, USA.

The focus species of this study is the sacoglossan sea slug *Elysia papillosa* (Figure 1), native to the greater Caribbean and Gulf of Mexico, and first formally described by Verrill (1901) [14,15]. This sea slug lives and feeds exclusively on green siphonaceous algae in the genus *Penicillus* [14,15,16,17]. The structure of *Penicillus* is advantageous for *E. papillosa* to feed on as the lack of thalli between septa allow for the sea slug to efficiently acquire the chloroplasts by puncturing an algal cell with their uniseriate radula and draining the contents [18,19]. Compared to other sacoglossans, *E. papillosa* is considered a short-term kleptoplast, able to retain functional chloroplasts for up to two weeks [12,20], in contrast to other species such as *Elysia chlorotica*, which feeds on *Vaucheria*, that can retain functional chloroplasts for up to nine months [6]. Although *E. papillosa* is one of the most abundant elysiid sea slugs in the Caribbean [15], it is understudied and much about its biology and microbiome remains unknown. Host-associated microbiomes play well-documented roles in the nutrition, immunity, and metabolic homeostasis of marine invertebrates. In sacoglossan gastropods specifically, bacterial communities associated with several *Elysia* species have been documented and their potential functional contributions to host biology are an active area of inquiry [21,22,23,24]. In kleptoplastic sacoglossans, the prolonged maintenance of functional chloroplasts in host tissue is metabolically demanding and likely places the isolated plastids under oxidative and proteotoxic stress. Whether associated bacteria could support this process by providing antioxidant capacity, protein quality control, or essential cofactors has not been investigated for *E. papillosa*, and forms one of the rationales of this study.

Previous studies on marine invertebrates, including sea slugs, have revealed the make-up of a variety of species’ microbiomes; however, the function of these microbes to the host is often unknown. When investigating the microbiome of sacoglossans, the function of microbes present may reveal possible mutualistic relationships occurring that could assist with the process of chloroplast collection and retention. In the genus *Elysia*, microbiomes of four species have previously been documented including *Elysia crispata* [21], *Elysia chlorotica* [22], *Elysia rufescens* [23], and *Elysia viridis* [24]. These previous studies have reported intraspecific variation in the composition of bacterial communities among sea slug populations sampled from identical environments, as well as between other environments and laboratory-reared individuals. Additionally, the microbial assemblages associated with the mucus secreted by sea slugs have been shown to differ from those associated with the whole organisms, likely due to the secretion of metabolites used for defense [23]. The differences among individuals of a singular species are interesting given the dietary specificity found in the *Elysia* genus. For example, *E. chlorotica* feeds almost exclusively on two species of the algae *Vaucheria* [25] and *E. rufescens* specializes on *Bryopsis* [26,27]. On the other hand, *E. crispata* is polyphagous, found to feed on multiple algal taxa including *Halimeda*, *Penicillus*, *Bryopsis*, and several others [28,29]. Dietary variation amongst species likely contributes to the observed differences in bacterial community composition and influences the duration of functional chloroplast retention.

The main objective of this study was to analyze the microbial community associated with *E. papillosa.* Specifically, we analyzed the bacterial diversity in this sea slug and compared these bacterial communities with communities identified from other sacoglossan species listed above to identify similarities between kleptoplastic sacoglossans. Such bacteria may serve as important symbionts for these slugs and might play a supportive role in kleptoplasty. In addition, another objective was to identify other potential symbiotic relationships, unrelated to kleptoplasty, between the bacterial communities and the sea slug.

## 2. Methods

### 2.1. Sample Collection and Preservation

Stalks of algae *Penicillus capitatus* that commonly have *Elysia papillosa* inhabiting them were collected by hand on snorkel in September 2021 at Sunset Beach in Tarpon Springs, FL, USA (28°8.71 N, 82°47.438 W). This shallow water site has a known population of *E. papillosa* and has been previously used in several other studies [12,14,16,17]. Algae was collected at low tide during slightly turbid conditions due to rainstorms throughout the day. The stalks of algae were stored in ambient seawater from the field site and transferred back to the laboratory at the University of Tampa where dissecting microscopes were required to locate and identify *E. papillosa*. Within four hours of algae collection from the field site, four specimens of *E. papillosa* were individually preserved in 40 mL of 70% ethanol and stored at 4 °C until extractions.

### 2.2. DNA Extraction and Shotgun Sequencing

One sample included the entire body of an individual preserved sea slug that was utilized in DNA extractions. Specimens ranged from 7 to 11 mm in body length. Body weight was not recorded at the time of collection. Animals were preserved in 70% ethanol within four hours of collection on 9 September 2021, stored at 4 °C, and DNA extractions were performed on 16 November 2021, giving a storage period of approximately 10 weeks. Each slug was processed whole using the Zymo Research Quick-DNA Mini-Prep kit (Tustin, CA, USA) following the manufacturer’s soft tissue protocol, without prior dissection. As a result, the dataset represents both surface-associated and internally associated bacteria, and we were unable to separate microbial communities by tissue compartment given the small body size of the specimens. No environmental controls (seawater, sediment, or algal microbiome samples) were collected alongside the specimens; host-associated and environmentally acquired bacteria therefore cannot be distinguished in this dataset. The relatively low DNA yields likely reflect the small body size of the specimens and the use of 70% ethanol preservation, which is not the optimal concentration for microbiome preservation. Higher concentrations (95–100%) are generally recommended as they penetrate tissues more rapidly and better inhibit DNase activity and may also introduce some bias in community composition compared to DESS-based preservation methods [30,31,32]. This likely contributed to the low DNA yields in Table 1 and may have affected the proportion of reads that could be classified. The four samples of isolated DNA from *E. papillosa* were sent to MRDNA in Shallowater, TX, USA, for shotgun metagenomic sequencing. Briefly, the libraries were prepared from 30 to 50 ng DNA using Illumina DNA Prep, (M) Tagmentation library preparation kit (Illumina, San Diego, CA, USA) following the manufacturer’s user guide. The samples underwent the simultaneous fragmentation and addition of adapter sequences. These adapters are utilized during a limited-cycle PCR in which unique indices were added to the sample. Following the library preparation, the final concentration of the libraries (Table 1) was measured using the Qubit^®^ dsDNA HS Assay Kit (Life Technologies, Carlsbad, CA, USA), and the average library size (Table 1) was determined using the Agilent 2100 Bioanalyzer (Agilent Technologies, Santa Clara, CA, USA). The libraries were then pooled in equimolar ratios of 0.6 nM, and sequenced paired-end for 300 cycles using the NovaSeq 6000 system (Illumina). Library preparation, pooling, and Illumina sequencing were carried out by MRDNA (Shallowater, TX, USA). All downstream bioinformatics work was performed by the authors, including quality control with FastQC v0.12.1, host read filtering with deacon v0.10.0, taxonomic classification with Centrifuger v1.0.11-r269, functional annotation with SqueezeMeta v1.7.2, diversity analyses, and figure generation.

### 2.3. Quality Control, Host Filtering, Taxonomic Classification, Functional Annotation, and Diversity Analysis

Sequences were quality checked using FastQC and all passed the adapter contamination and per base quality checks. Host sequences were filtered out from the data using the deacon program [33] against the closely related *Elysia chlorotica* genome as a reference for *E. papillosa* is not available (Genbank accession GCA_003991915.1). The resulting sequences were classified with Centrifuger [34] using default parameters against the Genome Taxonomy Database (GTDB r226) [35] and quantified using the Centrifuger-quant program. Because the GTDB r226 database contains only bacterial and archaeal genomes with no organellar sequences, any residual chloroplast reads cannot be assigned to a database entry and simply fall into the unclassified fraction, making an explicit plastid filtering step unnecessary. To verify this, we mapped raw reads from all four samples against the *Penicillus capitatus* chloroplast genome using Bowtie2 v2.5; mapping rates were 0.63%, 0.02%, 0.19%, and 0.00% for S1–S4 respectively, confirming negligible plastid contamination. As a further check, Cyanobacteria, the closest bacterial relatives of chloroplasts, were present at only 0.11–0.45% relative abundance across all four samples, consistent with minimal plastid misclassification. Rarefaction curves were produced to determine biodiversity of samples at the phylum and species level using the vegan R package v2.7-1 [36]. Rarefaction curves were generated from the Centrifuger-quant rank-specific numReads output. Because the sequencing data were paired-end, Table 2 reports classified reads as individual reads, whereas Centrifuger-quant reports classified fragments/read pairs. Rarefaction was therefore performed using rank-specific classified fragment counts: phylum-rank counts for the phylum rarefaction curve and species-rank counts for the species rarefaction curve. This explains why sample S4, despite having 1,934,254 classified reads in Table 2, contributes 814,627 species-level classified fragments to the species rarefaction analysis. Alpha diversity was calculated as Shannon entropy H’ using species-rank classified fragment counts. Beta diversity was calculated as Bray–Curtis dissimilarity from species-level relative abundances using SciPy v1.17 [37]. Sequences from samples were also mined for potential functional information using the SqueezeMeta pipeline [38]. The SqueezeMeta pipeline assembled reads into contigs, predicted Open Reading Frames (ORFs) with Prodigal [39], and searched the genes against the databases KEGG [40], COG [41], and PFAM [42] to assign functional information. The pipeline provided gene abundance values as Transcripts Per Million (TPM) and only bacterial assignments were retained in ORF filtering. Bacterial ORFs were categorized into functional groups by examining KEGG, COG, and PFAM accessions relevant to kleptoplast maintenance. The full annotated ORF table is provided as Supplementary Table S2. The relative gene abundance per functional category was calculated as a summed average TPM across the four samples.

## 3. Results

**Figure 2:**
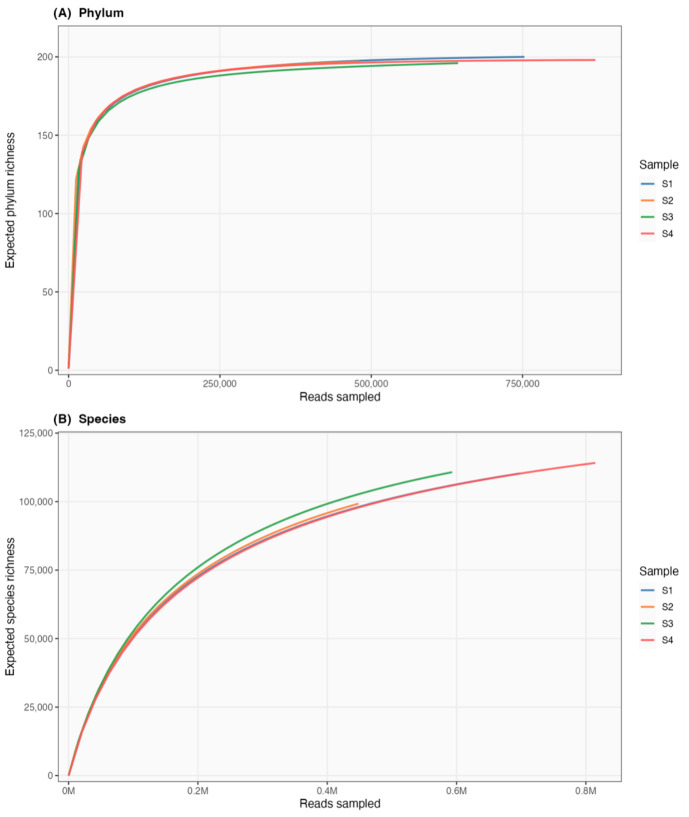
Rarefaction curves of number of reads sampled for the expected phylum richness (**A**) and expected species richness (**B**) for the four *Elysia papillosa* samples.

Across the four samples of *E. papillosa*, the number of classified sequencing reads from the number of reads after removing host sequences of the closely related *E. chlorotica* remained around one million (Table 2). Further, from these samples only 10–11% of sequencing reads were assigned a taxonomic classification (Table 2), consistent with expectations for environmental shotgun metagenomes. Reference databases such as the GTDB capture only a fraction of environmental microbial diversity and the remainder, often called microbial “dark matter”, lacks genomic representation and cannot be classified [30]. From the classified reads the expected richness of bacterial phyla plateaued around 500,000 reads sampled across all four *E. papillosa* samples indicating that the sequencing depth accounted for phyla diversity (Figure 2A). In contrast, the expected species richness curves for all four samples did not plateau suggesting that the richness on the species level was not fully captured, and more sequencing would reveal additional taxa (Figure 2B). Although GTDB includes both bacterial and archaeal genomes, archaea were detected at low relative abundance across all four samples, ranging from 0.10% to 1.95%. Therefore, GTDB r226 defined the reference space for classification, but the taxa reported here represent only those detected in the *E. papillosa* samples after classification and filtering. As the study was designed around bacterial diversity and archaea were consistently low in abundance, they are not discussed further.

Alpha diversity was high and consistent across all four samples, with Shannon entropy H’ ranging from 10.80 to 10.93. Beta diversity analysis showed moderate Bray–Curtis dissimilarity between all pairwise sample comparisons (range 0.26–0.30), suggesting some inter-individual variation within this small sample from a single population.

### 3.1. Phylum Relative Abundance

**Figure 3:**
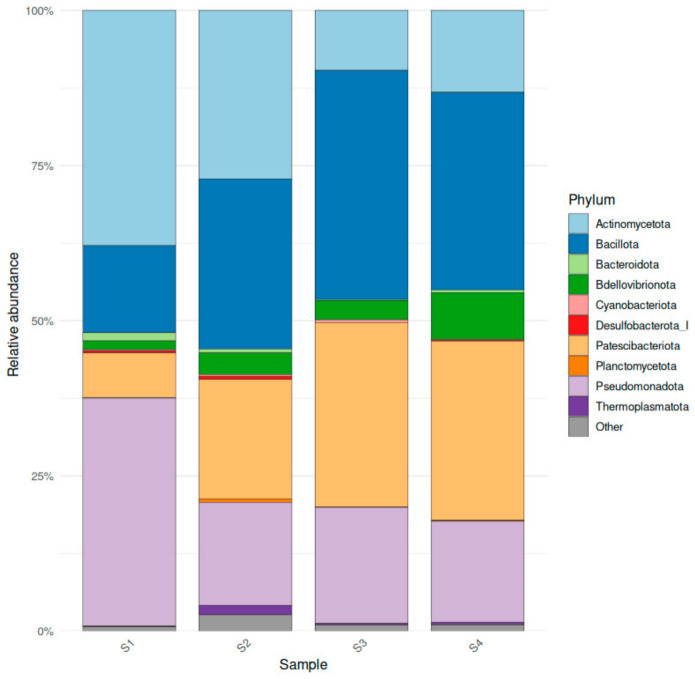
Relative abundance of bacterial phyla classified in the four samples of *Elysia papillosa* including the ten most abundant phyla and any remaining lower-abundant phyla grouped into the Other category.

Nine of the most relative abundant bacterial phyla were present in all four samples, which included Actinomycetota, Bacillota, Bacteroidota, Bdellovibrionota, Cyanobacteria, Pateschibacteriota, Planctomycetota, Pseudomonadota, and Thermoplasmatota. The phylum Desulfobacterota_I was the only one that was not recorded in all samples and was absent from sample 3 (Figure 3). All samples were dominated by Bacillota, Pseudomonadota, Actinomycetota, and Patescibacteriota with an average relative abundance range of 21–27%. The remaining phyla Bdellovibrionota, Bacteroidota, Thermoplasmatota, Desulfobacterota_I, Cyanobacteria, and Planctomycetota had relatively smaller relative abundances amongst the samples, ranging from 0.22 to 3.88%. Other phyla with lower relative abundances that were not included in the ten most abundant reported an average of around 1% across the four samples.

### 3.2. Genus Relative Abundance

**Figure 4:**
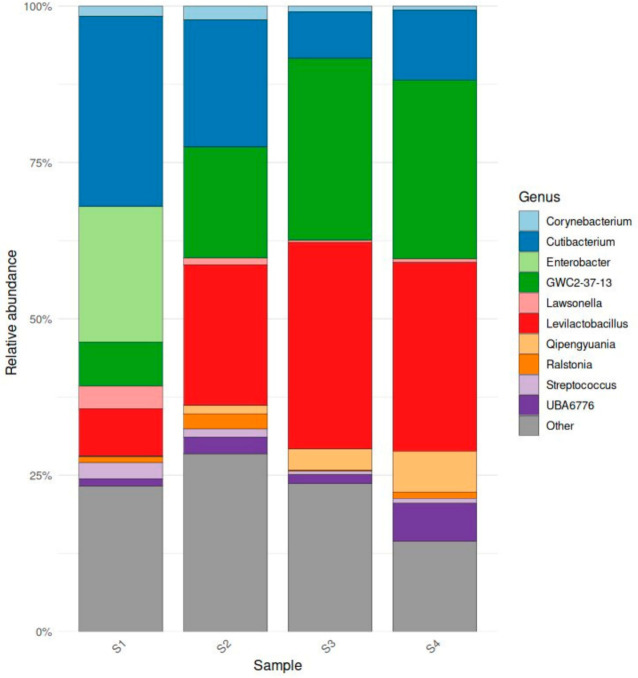
Relative abundance of bacterial genera classified in the four samples of *Elysia papillosa* including the ten most abundant genera; remaining lower-abundance genera are grouped into the Other category.

At the genus level, *Levilactobacillus* was the most abundant genus across all samples, averaging 23.3% relative abundance, followed by *GWC2-37-13* (20.6%) and *Cutibacterium* (17.3%) (Figure 4). *Enterobacter* was the fourth most abundant genus overall (average 5.4%) but was detected almost exclusively in sample S1 (21.7%), being absent from the other three individuals. *Qipengyuania* (2.8%), *UBA6776* (2.8%), and *Lawsonella* (1.4%) were present at lower abundances across most samples. The genus-level composition reflects the phylum-level patterns, with Bacillota and Actinomycetota dominated by *Levilactobacillus* and *Cutibacterium* respectively, and Pseudomonadota distributed across several genera including *Qipengyuania*, *UBA6776*, *Ralstonia*, and *Caldimonas* (Supplementary Table S1; Figure 4).

### 3.3. Species Relative Abundance

**Figure 5:**
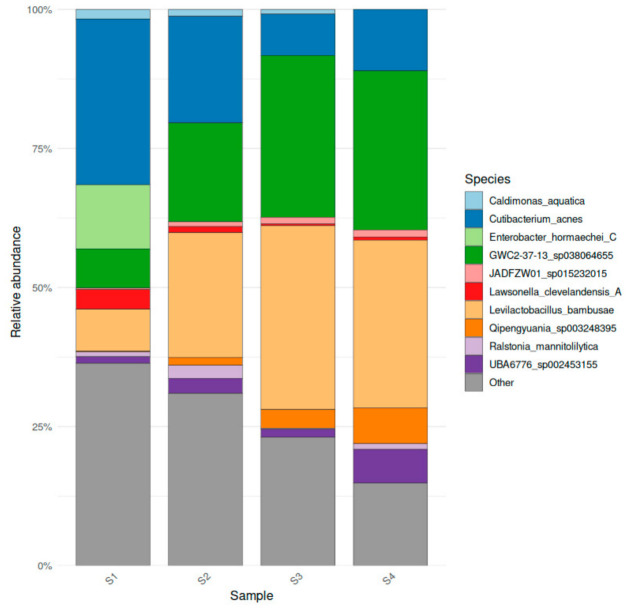
Relative abundance of bacterial species classified in the four samples of *Elysia papillosa* including the ten most abundant species and any remaining lower-abundant species grouped into the Other category.

The ten species with the highest relative abundance in the *E. papillosa* samples were *Caldimonas aquatica*, *Cutibacterium acnes*, *Enterobacter hormaechei C*, *GWC2-37-13_sp038064655*, *JADFZW01_sp015232015*, *Lawsonella clevelandensis A*, *Levilactobacillus bambusae*, *Qipengyuania_sp003248395*, *Ralstonia mannitolilytica*, and *UBA6776_sp002453155* (Figure 5). All species were reported in the samples excluding *Caldimonas aquatica*, which was not reported in sample four, and *Enterobacter hormaechei C,* which was notably only identified in sample one. These ten species represent the most abundant species-level assignments shown in Figure 5, with the remaining species-level reads distributed across a large number of lower-abundance taxa in the Other category. Of the reads that received a classification, 87–89% were assigned at the species level; however, since only 10–11% of all sequencing reads were classified, approximately 9% of total reads obtained a species-level assignment. This is consistent with the limited genomic representation of marine environmental bacteria in current reference databases. The species that dominated the samples with an average relative abundance ranging from 16 to 23% were *Levilactobacillus bambusae*, *GWC2-37-13_sp038064655*, and *Cutibacterium acnes*. Other lower-abundance species that were present in all samples, such as *UBA6776_sp002453155*, *Qipengyuania_sp003248395*, *Lawsonella clevelandensis A*, *Ralstonia mannitolilytica*, and *JADFZW01_sp015232015*, ranged from approximately 0.9% to 2.8% average relative abundance.

### 3.4. Core Microbiome

To determine similarities in bacterial phyla and species amongst samples a core microbiome was defined as taxa that were present in all four *E. papillosa* samples with a relative abundance of 0.1%. At the phylum level the core microbiome consisted of seven taxa from the ten most abundant discussed above and included Bacillota, Pseudomonadota, Actinomycetota, Patescibacteriota, Bdellovibrionota, Bacteroidota, and Cyanobacteria (Table 3). On the other hand, at the species level eight of the ten most abundant taxa were identified to make-up the core microbiome (Table 4). Two bacterial species from the core microbiome belonged to each of these phyla: Pseudomonadota (i.e., *Ralstonia mannitolilytica, Qipengyuania sp003248395*), Actinomycetota (i.e., *Cutibacterium acnes*, *Lawsonella clevelandensis*), and Bdellovibrionota (i.e., *UBA6776 sp002453155*, *JADFZW01 sp015232015*). Other representative species were *Levilactobacillus bambusae* and *GWC2-37-13 sp038064655* with their respective phyla being Bacillota and Patescibacteriota.

### 3.5. Functional Gene Groups Present

**Figure 6:**
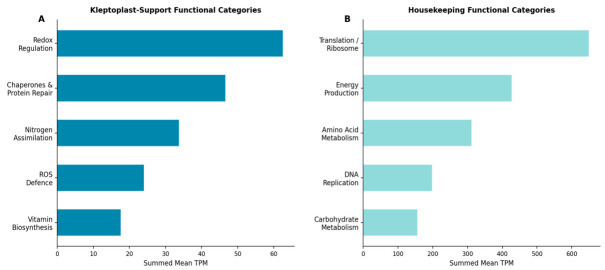
**(A**): Five functional categories relevant to kleptoplast support showing summed mean TPM across the four *E. papillosa* samples. (**B**): Five major housekeeping functional categories (translation/ribosome, energy production, amino acid metabolism, DNA replication, and carbohydrate metabolism) shown for comparison, calculated by the same method from the same dataset. The five kleptoplast-support categories combined account for 184.68 summed mean TPM, representing 2.67% of total bacterial gene abundance across 3737 annotated bacterial ORFs.

Five functional categories were selected to provide an overview of the number of genes present in the microbiome of *E. papillosa* that may play a role in sustaining kleptoplasty within the sea slug microbiome. From these focused categories the summed average of TPMs for the most abundant genes to least abundant in the sea slugs were redox regulation, chaperones and protein repair, nitrogen assimilation, reactive oxygen species (ROS) defense, and vitamin biosynthesis (Figure 6).

## 4. Discussion

This study aimed to analyze the abundance of bacterial species associated with the sacoglossan sea slug *Elysia papillosa* through shotgun metagenomic analysis to hypothesize reasonings for the presence of highly abundant species in the microbiome based on functional gene groups and the supporting literature. The majority of sequenced reads fell under the unclassified category, which may be associated with the bacterial specific database selected (i.e., GTDB r226) that either had organisms not represented or the reads belonged to fungi and viruses that were not classified, in turn resulting in a lower classification rate. Another explanation for this phenomenon may be due to our sample preservation method using 70% ethanol that could have resulted in DNA degradation and lower amounts of classified reads. A range of studies investigating preservation methods for biological samples have reported higher-quality DNA for methods such as higher-concentration ethanol [31,32,43] or DESS, a mixture of DMSO, EDTA, and NaCl [44]. For the current study 70% ethanol was an affordable and efficient preservation method; while it has been utilized in other mollusk studies [45,46], the different preservation methods and possible impacts on microbiomes for sea slug samples and other mollusks should be investigated further.

### 4.1. Abundance of Bacterial Phyla and Associated Species

The dominant Bacillota signal was driven primarily by *Levilactobacillus bambusae*, which averaged 23.3% relative abundance across all four samples and was the single most abundant species detected. *Levilactobacillus* is a lactic acid bacterium originally described from bamboo shoot fermentation, and its high abundance in *E. papillosa* is notable for a marine organism. Lactic acid bacteria, including members of the former genus *Lactobacillus*, have been evaluated as probiotics in shrimp aquaculture, where they may contribute to host health through immune modulation and competitive exclusion of pathogens [47]. Other microbial studies on *Elysia* species have reported Bacillota, although generally at lower abundances than observed here [21,22,23], and Bacillota were not recorded in one previous study [24]. Because those studies used 16S rRNA sequencing and different preservation approaches, percentages are not directly comparable. The high abundance of *L. bambusae* may partly reflect proximity of the collection site to freshwater input from the Anclote River, since Bacillota can be common in freshwater-influenced environments; however, its consistent presence across all four individuals makes a purely environmental explanation less convincing and leaves open the possibility of a commensal or host-associated role. It is also worth noting that several species detected in this study are primarily known from clinical settings, including *Cutibacterium acnes* and *Lawsonella clevelandensis A*. This likely reflects biases in available reference genomes, where environmental reads may be assigned to the nearest characterized relative rather than the true organism present. This should be kept in mind when interpreting species-level assignments [48,49].

Pseudomonadota (formerly Proteobacteria) was the second most dominant phylum by average in the samples of *E. papillosa* (Figure 3). This phylum is one of the most abundant and diverse bacteria [22]. Of the ten most abundant species found (Figure 5), four species that were in all samples analyzed belonged to the Pseudomonadota phylum, including *Caldimonas* aquatica, *UBA6776_sp002453155*, *Ralstonia mannitolilytica*, and *Qipengyuania_sp003248395*. *R. mannitolilytica* has been isolated from insect guts and shown to be able to degrade cellulose [50]. In particular, *Qipengyuania* has been isolated from marine environments and can produce carotenoids [51] which may aid *E. papillosa* in light harvesting or in photoprotection. Members of *Caldimonas* have been shown to utilize cellobiose and to produce amylase [52,53], which may help *E. papillosa* in carbohydrate metabolism. An additional species from the Pseudomonadota phylum that was reported only in sample one at 11.5% relative abundance was *Enterobacter hormaechei* C. Considering *E. hormaechei* is commonly studied as a pathogen in humans, there is limited research of its impact on mollusks and therefore may not indicate a presence of disease for this sea slug individual. In fact, a study on *E. hormaechei* isolated from the gut of flathead grey mullet reported this bacteria to be a potential probiotic to the host suggesting this bacteria needs to be investigated more for marine organisms [54].

The next two most abundant phyla were Actinomycetota (formerly Actinobacteria) and Pastescibacteriota. Similar to Bacillota, the phyla Actinomycetota tend to be more prevalent in freshwater environments [22,55]. Compared to the other studies examining sacoglossan microbiomes, *E. papillosa* in some cases had over double the abundance of species from Actinomycetota than other *Elysia* species, but again these studies were conducted using 16S rRNA sequencing [22,23]. Furthermore, these phyla dominated the microbiome of *E. viridis* in addition to Bacteroidota and may be indicative of freshwater influence as the site collection was near a river output [24]. Freshwater inputs at the collection site is one possible explanation for the high abundance of Actinomycetota species. Although members of Actinomycetota were not as abundant in other *Elysia* species and their presence may be environmentally driven, this does not rule out the possibility that some members of this group function as symbionts in *E. papillosa*. For example, Devine et al. [22] identified in the microbiome of *E. chlorotica* members of the family Propionibacteriaceae within the phylum Actinomycetota, a bacterial group that includes species capable of producing vitamin B12, which is an essential nutrient for the survival of sea slugs during periods of algal deprivation. In this study, the species *Cutibacterium acnes* from the family Propionibacteriaceae was present in all *E. papillosa* samples. Indeed, *C. acnes* was isolated from the gut of the marine polychaete *Capitella teleta* and was reported to be a member of the worm’s core functional microbiota [48]. The same is possible in *E. papillosa* where this bacterium might provide the slug with nutrients such as vitamin B12 for growth and survival. The other member of Actinomycetota found in the *E. papillosa* samples, *Lawsonella clevelandensis* A is a non-marine human pathogen [49] possibly introduced into the water by human inputs. The introduction of this species could likely be due to the collection site that is a few miles south of the Anclote River. This creek with a spring-fed water source could alter the salinity at the site and affect the microbes that can inhabit the area [56].

The *E. papillosa* samples also contained members of the phylum Bacteroidota (Figure 3) with the most abundant species reported with uninformative names: *GWC2-37-13_sp038064655* and *JADFZW01_sp015232015* (Figure 5). Nevertheless, members of Bacteroidota have been shown to cause disease outbreaks in their eukaryotic marine hosts [57] and it is possible that they are pathogens for the sea slug. However, members of Bacteroidota can also degrade polymers such as cellulose, agar, and chitin [58]. Future studies investigating the function of these bacterial species in marine organisms would help elucidate their potential role in *E. papillosa*.

Other lower-abundant phyla included Desulfobacterota I, Cyanobacteria, Thermoplasmatota, Planctomycetota, and Bdellovibrionota. The phylum Desulfobacterota I was also found in the *E. papillosa* samples. Members of Desulfobacterota have also previously been found in the deep-sea shrimp Rimicaris kairei from the Indian Ocean [59]. Sulfur-oxidizing endosymbionts have been found in the gutless marine tubeworm *Riftia pachyptila* [60], and members of Desulfobacterota are known to carry out sulfur cycling in marine environments. In addition, Desulfobacterota can also produce carotenoids [61] which may aid *E. papillosa* in the photosynthetic process and may also protect against radiation, desiccation, and oxidation. The Cyanobacteria detected here are free-living bacteria classified against the GTDB prokaryote-only database and are distinct from the algal chloroplasts of chlorophyte origin retained through kleptoplasty. Cyanobacteria are involved in symbiotic relationships with seagrasses, sponges, ascidians, etc. [62]. They produce bioactive compounds which can be photoprotective and contribute to anti-grazing. Planctomycetota degrade high-molecular-weight sugars in phototrophs like algae [63] and may serve this purpose in *E. papillosa* as well. It is unclear what role the archaea Thermoplasmatota may play in *E. papillosa*, but archaea have been shown to carry out ammonia oxidation in marine hosts in coral reefs [64]. Bdellovibrionota members have been found in marine waters and are obligate predators who feed on Gram-negative bacteria [65]. The class Oligoflexia has chitinase genes to degrade chitin and this is thought to serve as a defense against eukaryotic (fungal) pathogens. While Bdellovibrionota is likely not a symbiont in *E. papillosa*, it brings up the intriguing possibility that this bacteria may indirectly help the sea slug defend against fungal pathogens which have chitin in their cell walls.

Notably, the bacterial family Flavobacteriaceae was detected at low abundance in our samples. This family has also been reported in the sea slug *E. viridis* [24], and certain members are known to synthesize carotenoids [66] and zeaxanthin [67]. Thus, Flavobacteriaceae associated with *E. papillosa* may contribute to carotenoid production, potentially shielding the host from oxidative stress and providing photoprotective benefits.

### 4.2. Potential Functional Benefits of Genes Present

Genes related to redox regulation within the present bacteria may support the harbored chloroplasts sacoglossan sea slugs acquire for nutrition. The presence of thioredoxins, ferredoxins, and glutaredoxins were found which are essential for maintaining the activity of enzymes that are used in photosynthesis [68]. The presence of these genes is consistent with a potential role in supporting photosynthetically active plastids, though this remains speculative without functional data. Since photosynthesis produces reactive oxygen species, the presence of genes encoding catalase–peroxidase, superoxide dismutase, and peroxidases is consistent with a role in protecting plastid membranes and proteins from oxidative stress, though functional activity remains to be demonstrated [69,70]. The presence of DnaK and GroEL/ES system chaperones and Clp proteases raises the possibility that bacteria could contribute to protein repair and turnover under stress conditions. These systems may help to mitigate the stress that the isolated plastids are under [71]. Genes for the biosynthesis of riboflavin and thiamin were also found and may provide the essential cofactors required for the process of photosynthetic electron transport [65,72]. Additionally, the presence of GS-GOGAT (Glutamine Synthetase–Glutamate Synthase) genes may mean that the bacteria have the potential to provide some reduced nitrogen for plastid protein and chlorophyll synthesis [73].

Pseudomonadota were among the most abundant phyla in all four samples, and may contribute to the functional landscape given their known roles in redox chemistry, vitamin biosynthesis, and nitrogen metabolism in other host-associated systems. The harboring of chloroplasts within the tissues of *E. papillosa* increases the risk of host cell degradation due to oxidative damage from the production of reactive oxygen species through photosynthetic processes [8]. Therefore, the presence of genes for detoxification of reactive oxidative species is crucial to protecting host cells as well as the functionality of the harvested chloroplasts that support *Elysia* nutrition [8]. Additionally, it has been shown that some marine host-associated microbial communities enhance the metabolism of their host by supplying vitamins, antioxidants, and nitrogen in a reduced form [74,75,76]. While the exact role of these microbiota in photosynthetic sea slugs remains unclear, the presence of genes from Pseudomonadota may suggest metabolic support to the host by extending the longevity of harbored chloroplasts through alleviating oxidative stress and nutrient limitation.

Aside from support for plastids, the bacterial community encodes genes that could possibly benefit *E. papillosa* more directly. Pathways for essential amino acid biosynthesis were found, which raises the possibility that bacteria could supplement host nutrition, analogous to nutritional endosymbionts of insects [77,78]. Genes for iron acquisition including transporters for both ferrous (FeoB) and ferric iron (AfuA) were also present and may help to scavenge iron from the environment for use in iron–sulfur clusters [79]. Vitamin B biosynthesis genes including those of riboflavin, folate, and biotin were also found. These are cofactors that animals are not able to synthesize [80] and so may help the sea slug in various metabolic processes. Urea recycling genes that encode urease and urea transporters were also found and have the potential to recover nitrogen from metabolic waste and possibly improve the nitrogen economy of the sea slug [81]. Also present were genes for the transport of glycine betaine which indicate a further role in osmotic adaptation to the marine environment [82].

### 4.3. Future Directions

Due to the specialization of *E. papillosa* feeding on *Penicillus*, an analysis of this algae’s microbiome is necessary to fully understand the relationship between the two organisms and to assist with differentiating between the origin of bacteria found in our samples as it may be likely that some may be from the algae due to their close association. It is possible that the sea slug receives more benefit than simply a source of chloroplasts, as seen in a study of *Elysia rufescens* and its food source [83]. The algae *Bryopsis* sp. was found to host a bacterium for protection that produces kahalalides which can be toxic to herbivores; however, *E. rufescens* is able to feed on this alga regardless of the toxins [83]. Furthermore, *E. rufescens* received from *Bryopsis* not only chloroplasts but also accumulated the kahalalides for protection from potential predators. In addition to *E. rufescens*, *Elysia grandifolia* and *Elysia ornata*, also found to feed on *Bryopsis*, had kahalalides isolated from them [26,84,85]. The findings described above have been important to the medical community as the specific kahalalide F (KF) that is found to be associated with *Bryopsis* and some *Elysia* sea slugs can cause cell death and is being used in drug trials to target cancer cells [86,87]. Overall, these interesting interactions detailed for other *Elysia* species reveal the importance of further analysis of the specific dietary source of *E. papillosa* and other sacoglossan species. Given these findings, we also searched all four *E. papillosa* samples for *Candidatus Endobryopsis kahalalidefaciens*, the bacterium responsible for kahalalide production in *Bryopsis*-feeding *Elysia* species. Three independent approaches were used: taxonomic classification with Centrifuger against GTDB, direct mapping of raw reads against the *Ca. Endobryopsis kahalalidefaciens* genome (JGI) using Bowtie2 v2.5, and a search of the SqueezeMeta functional annotation output for iterative PKS/NRPS machinery including adenylation domains, condensation domains, and ketosynthase modules. All three returned negative results, with 0.00% read mapping across all four samples. The 47 acyl-carrier-protein-related ORFs detected in the annotation were all standard fatty acid synthesis genes (fabG, fabH, fabI, fabA, fabZ, acpP), which are unrelated to kahalalide biosynthesis despite sharing the ACP domain with polyketide synthases. This is consistent with the dietary biology of *E. papillosa*, which feeds exclusively on *Penicillus* rather than *Bryopsis* and would not be expected to harbor this organism.

## 5. Conclusions

This study presents the first shotgun metagenomic characterization of the bacterial community of *Elysia papillosa*, a short-term kleptoplastic sacoglossan from the eastern Gulf of Mexico. Four whole-body specimens (7–11 mm) from a single population shared a broadly consistent bacterial community dominated by Bacillota, Pseudomonadota, Actinomycetota, and Patescibacteriota, with *Levilactobacillus bambusae*, *GWC2-37-13_sp038064655*, and *Cutibacterium acnes* the most abundant species. Alpha diversity was high and similar across individuals (Shannon entropy H’ 10.80–10.93), and beta diversity showed moderate pairwise variation among individuals, consistent with some inter-individual variation within this small sample from a single population. Functional gene analysis identified bacterial potential for redox regulation, ROS defense, chaperone-mediated protein repair, vitamin biosynthesis, and nitrogen assimilation, representing 2.67% of total bacterial gene abundance across 3737 annotated ORFs. These are categories that could plausibly support kleptoplast stability, though the data reflect genetic potential only and further work is needed to establish any functional role. Searches for *Candidatus Endobryopsis kahalalidefaciens* and its PKS/NRPS biosynthetic machinery returned no hits across all four samples by three independent methods, consistent with *E. papillosa*’s exclusive diet of *Penicillus* rather than *Bryopsis*. The main limitations of this study are the small sample size from a single site and timepoint, whole-body extraction without anatomical dissection, and the lack of environmental controls, all of which limit ecological inference. Nevertheless, this dataset provides a useful baseline for *E. papillosa* and a reference point for future comparative work across the Sacoglossa. Future studies should incorporate microbiome sampling of *Penicillus capitatus*, ambient seawater, and sediment from the same site, along with larger and more geographically diverse samples and transcriptomic or proteomic approaches to move beyond genomic potential.

## Figures and Tables

**Table 1:** Summary of extracted genomic DNA of the four *Elysia papillosa* samples including DNA concentration, final library DNA concentration, and average library size.

Sample ID	DNA Concentration (ng/µL)	Final Library DNA Concentration (ng/µL)	Average Library Size (bp)
S1	1.49	27.60	731
S2	2.14	26.80	903
S3	9.04	29.20	748
S4	3.78	27.40	662

**Table 2:** Summary of sequencing reads for *E. papillosa* samples including the total number of reads, reads remaining after filtering host sequences against closely related *E. chlorotica*, and number of classified reads.

Sample ID	Total Number of Reads	Number of Reads After Host Filtering	Number of Classified Reads	Percentage of Classified Reads
S1	17,255,060	15,163,120	1,675,334	11.05%
S2	11,336,748	10,016,608	1,062,946	10.61%
S3	14,470,346	12,629,384	1,438,062	11.39%
S4	21,984,086	19,057,154	1,934,254	10.15%

**Table 3:** Core phyla taxa present in all *E. papillosa* samples with a minimum relative abundance of 0.1%.

	Sample Identification				
Phylum	S1	S2	S3	S4	Average
Bacillota	14.02%	27.43%	36.93%	31.84%	27.55%
Pseudomonadota	36.74%	16.59%	18.67%	16.30%	22.07%
Actinomycetota	37.90%	27.16%	9.66%	13.19%	21.98%
Patescibacteriota	7.23%	19.24%	29.69%	28.85%	21.25%
Bdellovibrionota	1.43%	3.52%	3.07%	7.52%	3.89%
Bacteroidota	1.32%	0.59%	0.18%	0.46%	0.64%
Cyanobacteria	0.18%	0.27%	0.45%	0.11%	0.25%

**Table 4:** Core species taxa present in all *E. papillosa* samples with a minimum relative abundance of 0.1%.

	Sample Identification				
Species	S1	S2	S3	S4	Average
*Levilactobacillus bambusae*	7.57%	22.50%	33.03%	30.20%	23.32%
*GWC2-37-13 sp038064655*	7.04%	17.78%	29.09%	28.61%	20.63%
*Cutibacterium acnes*	29.81%	19.17%	7.45%	11.04%	16.87%
*UBA6776 sp002453155*	1.15%	2.65%	1.44%	6.06%	2.83%
*Qipengyuania sp003248395*	0.13%	1.35%	3.38%	6.39%	2.81%
*Lawsonella clevelandensis A*	3.53%	1.06%	0.33%	0.56%	1.37%
*Ralstonia mannitolilytica*	0.86%	2.39%	0.18%	1.03%	1.12%
*JADFZW01 sp015232015*	0.26%	0.86%	1.17%	1.27%	0.89%

## Data Availability

The sequence data have been deposited in the SRA under BioProject ID: PRJNA1258580.

## Supplementary Materials

The following supporting information can be downloaded at: https://www.mdpi.com/article/10.3390/biom16060918/s1, Table S1: Taxonomic abundance summary; Table S2: Functional annotation output.
